# Hoodigogenin A from *Hoodia gordonii*

**DOI:** 10.1107/S1600536808022368

**Published:** 2008-07-31

**Authors:** Yatin J. Shukla, Frank R. Fronczek, Rahul S. Pawar, Ikhlas A. Khan

**Affiliations:** a Department of Pharmacognosy, School of Pharmacy, University of Mississippi, University, MS 38677, USA; b Department of Chemistry, Louisiana State University, Baton Rouge, LA 70803-1804, USA; c National Center for Natural Products Research, Research Institute for Pharmaceutical Sciences, University of Mississippi, University, MS 38677, USA

## Abstract

The title mol­ecule (systematic name: 12-*O*-β-tigloyl-3β,14β-dihydroxy­pregn-5-en-20-one), C_26_H_38_O_5_, isolated from aerial parts of *Hoodia gordonii*, has its steroid *A* and *C* rings in chair conformations, its *B* ring in a half-chair conformation, and its five-membered ring in an envelope conformation. The OH group at the *C*/*D* ring junction forms an intra­molecular hydrogen bond with the keto substituent. The OH group on the *A* ring forms an inter­molecular hydrogen bond with the tiglate C=O group, propagating [010] chains in the crystal structure.

## Related literature

For related literature, see: Allen (2002 ▶); Consumer Reports (2006 ▶); Etter (1990 ▶); MacLean & Luo (2004 ▶); Muller & Albers (2002 ▶); Nutrition Business Journal (2007 ▶); Pawar *et al.* (2007 ▶); Shin *et al.* (1990 ▶); Van Heerden *et al.* (1998 ▶).
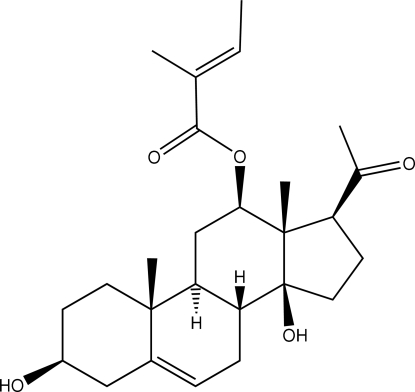


## Experimental

### 

#### Crystal data

C_26_H_38_O_5_
*M* *_r_* = 430.56
Orthorhombic, 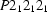

*a* = 7.6523 (9) Å
*b* = 10.6885 (12) Å
*c* = 27.705 (3) Å
*V* = 2266.0 (4) Å^3^
*Z* = 4
Mo *K*α radiation
μ = 0.09 mm^−1^
*T* = 100 K
0.30 × 0.27 × 0.05 mm

#### Data collection

Nonius KappaCCD (with an Oxford Cryosystems Cryostream cooler) diffractometer
Absorption correction: none
9377 measured reflections
2677 independent reflections
2156 reflections with *I* > 2σ(*I*)
*R*_int_ = 0.030

#### Refinement

*R*[*F* ^2^ > 2σ(*F* ^2^)] = 0.040
*wR*(*F* ^2^) = 0.100
*S* = 1.02
2677 reflections
287 parameters
H-atom parameters constrained
Δρ_max_ = 0.20 e Å^−3^
Δρ_min_ = −0.17 e Å^−3^

### 

Data collection: *COLLECT* (Nonius, 2000 ▶); cell refinement: *SCALEPACK* (Otwinowski & Minor, 1997 ▶); data reduction: *SCALEPACK* and *DENZO* (Otwinowski & Minor, 1997 ▶); program(s) used to solve structure: *SIR97* (Altomare *et al.*, 1999 ▶); program(s) used to refine structure: *SHELXL97* (Sheldrick, 2008 ▶); molecular graphics: *ORTEP-3* (Farrugia, 1997 ▶); software used to prepare material for publication: *SHELXL97*.

## Supplementary Material

Crystal structure: contains datablocks global, I. DOI: 10.1107/S1600536808022368/hb2752sup1.cif

Structure factors: contains datablocks I. DOI: 10.1107/S1600536808022368/hb2752Isup2.hkl

Additional supplementary materials:  crystallographic information; 3D view; checkCIF report

## Figures and Tables

**Table 1:** Hydrogen-bond geometry (Å, °)

*D*—H⋯*A*	*D*—H	H⋯*A*	*D*⋯*A*	*D*—H⋯*A*
O1—H10H⋯O5^i^	0.84	2.11	2.941 (3)	173
O2—H20H⋯O3	0.84	2.10	2.886 (2)	156

Symmetry code: (i) 
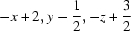
.

**Fig. 1.**
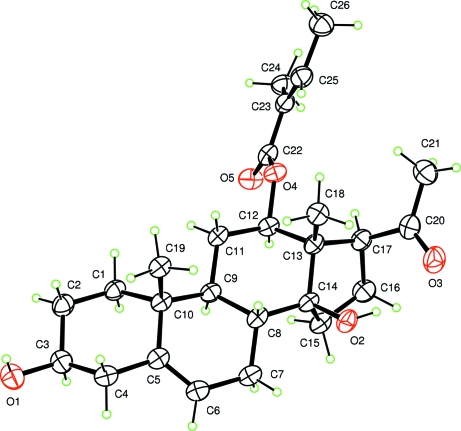
View of the molecular structure of (I), with displacement ellipsoids at the 50% level (spheres of arbitrary radius for the H atoms).

## supplementary crystallographic information

### Comment

*Hoodia gordonii*, a succulent plant, is one of the 18 species of *Hoodia* (Fam. Asclepiadaceae) that are indigenous to the summer rainfall regions of the Kalahari Desert in South Africa, Botswana and Namibia (Muller & Albers, 2002). In past few years, *Hoodia gordonii* has gained popularity as a weight-loss dietary supplement (Van Heerden *et al.*, 1998; Consumer Reports, 2006; Nutrition Business Journal, 2007; MacLean & Luo, 2004). As a part of our ongoing studies on *Hoodia*, we recently described isolation and characterization of 11 new oxypregnane glycosides (Hoodigosides A—K) along with P57AS3, the reported active oxypregnane glycoside from *H. gordonii* (Pawar *et al.*, 2007). These glycosides consist of the title compound, hoodigogenin A, (I) as the aglycone. (I) is an unique pregnane derivative, owing to the *cis*-fusion of rings C and D of the steroid skeleton and the tiglic ester functionality at C12, and is only reported so far from *H. gordonii*.

The preliminary structure of (I) was elucidated with the help of one-dimensional and two-dimensional NMR, and HRESI-MS methods. The relative configurations were established using the NOESY correlations, in which (I) was characterized as having β-OH groups at C3, and C14. The tiglic ester substitution at C12 and the acetyl side chain at C17 were assigned a β orientation as well (Pawar *et al.*, 2007). Here we report the crystal structure of (I) (Fig. 1), which confirms the relative configurations ascribed by the NMR studies.

The A and C rings (C1—C5, C10; C8, C9, C11—C14 respectively) have chair conformations, with endocyclic torsion-angle magnitudes in the range 42.2 (3) to 60.1 (2)°. The unsaturated B ring (C5—C10) has a half-chair conformation, with C8 displaced by 0.431 (2) Å and C9 by -0.373 (2) Å from the best plane of the other four C atoms. The five-membered D ring has an envelope conformation, with C14 at the flap position, displaced by 0.625 (2) Å from the best plane of the other four C atoms. The conformation of the C(O)Me group with respect to the main skeleton is defined by the torsion angle C16—C17—C20—O3, -37.5 (3)°, and the conformation of the tiglate substituent with respect to the skeleton by C11—C12—O4—C22, -102.8 (2)°. The tiglate is approximately planar, having a slight twist of -5.2 (3)° about its central bond (O4—C22—C23—C25).

The O2—H group forms an intramolecular hydrogen bond with C(O)Me carbonyl O3, making a discrete seven-membered ring, graph set S(7) (Etter, 1990). The O1—H group forms an intermolecular hydrogen bond with tiglate O5 (at 2-*x*, *y*-1/2, 3/2-*z*), thus making chains in the [0 1 0] direction (Table 1).

The Cambridge Structural Database (version 5.29, Nov. 2007; Allen, 2002) contains only one Δ^5^-pregnane steroid having O-substituents at C3, C12, and C14, refcode SENKUR (Shin *et al.*, 1990). SENKUR, like (I) has OH groups at C3 and C14, a benzoate at C12, and also OH groups at C8 and C17. The conformations of the A, B, and C rings in (I) and SENKUR are similar, with 18 endocyclic torsion angles differing by a mean value of 5.7°. The five-membered ring of SENKUR, however, has an envelope conformation with a different atom, C13 at the flap position. This may be a result of the fact that SENKUR has the opposite configuration at C17, with the C(O)Me substituent α oriented (Shin *et al.*, 1990).

### Experimental

Powdered aerial parts of *H. gordonii* were purchased from a commercial supplier. The plant material was authenticated by Vaishali Joshi by comparing with authentic sample of *H. gordonii* obtained from Missouri Botanical Garden Missouri, USA. A voucher specimen (Voucher No. 2799) has been deposited in the repository of The National Center for Natural Product Research. 4.75 kg of coarsely powdered *H. gordonii* was extracted by percolation with CHCl_3_ (4 × 4 *L*). These extracts were combined and concentrated to obtain a thick mass (402.1 g). The extract was dissolved in MeOH/H_2_O (95:5 v/v) and partitioned with hexane. The polar fraction (136 g) was subjected to VLC on silica gel (1500 g) by eluting with gradients of CHCl_3_/MeOH/H_2_O from 100:4:0.5 (3*L*), up to 90:12:0.5 (by increasing MeOH and reducing CHCl_3_, each by 1% increments), to generate eight sub-fractions.

The sub-fraction 2 was repeatedly chromatographed on a silica gel column using isocratic solvent systems of hexane: CHCl_3_ (2:98) and CH_2_Cl_2_ (100%) that led to isolation of the title compound as an amorphous solid. Compound (I) displayed a prominent blue spot on TLC upon spraying with anisaldehyde-H_2_SO_4_ reagent, followed by heating at 100 °C for 1–2 minutes. Further, (I) was dissolved in acetone:hexane (70:30 v/v), which upon standing at room temperature for 24 h yielded 150 mg of colorless crystals.  Colorless plates of (I) suitable for X-ray diffraction were obtained by recrystallization in hexane with few drops of acetone. The specific rotation of hoodigogenin A [α]^25^_D_ is +1.33 (c0.3, CHCl_3_). Detailed HRESI-MS and NMR data for (1) were described previously (Pawar *et al.*, 2007).

### Refinement

The absolute configuration of (I) could not be determined, and was chosen to agree with the accepted configuration of pregnane steroids (C3 S, C8 R, C9 S, C10 R, C12 R, C13 S, C14 S, C17 S): Friedel pairs were averaged before refinement.

The H atoms were placed in idealized positions (C—H = 0.95-0.99Å, O—H = 0.84Å) and thereafter treated as riding with *U*_iso_(H) = 1.2*U*_eq_(C) or 1.5U_eq_(methyl C, O).

### Crystal data

**Table:**

C_26_H_38_O_5_	*F*_000_ = 936
*M**_r_* = 430.56	*D*_x_ = 1.262 Mg m^−^^3^
Orthorhombic, *P*2_1_2_1_2_1_	Mo *K*α radiation λ = 0.71073 Å
Hall symbol: P 2ac 2ab	Cell parameters from 2446 reflections
*a* = 7.6523 (9) Å	θ = 2.5–26.6º
*b* = 10.6885 (12) Å	µ = 0.09 mm^−^^1^
*c* = 27.705 (3) Å	*T* = 100 K
*V* = 2266.0 (4) Å^3^	Plate, colorless
*Z* = 4	0.30 × 0.27 × 0.05 mm

### Data collection

**Table:**

Nonius KappaCCD (with an Oxford Cryosystems Cryostream cooler) diffractometer	2156 reflections with *I* > 2σ(*I*)
Radiation source: fine-focus sealed tube	*R*_int_ = 0.030
Monochromator: graphite	θ_max_ = 26.7º
*T* = 100 K	θ_min_ = 2.7º
ω and φ scans	*h* = −9→9
Absorption correction: none	*k* = −13→13
9377 measured reflections	*l* = −34→34
2677 independent reflections	

### Refinement

**Table:**

Refinement on *F*^2^	Secondary atom site location: difference Fourier map
Least-squares matrix: full	Hydrogen site location: inferred from neighbouring sites
*R*[*F*^2^ > 2σ(*F*^2^)] = 0.040	H-atom parameters constrained
*wR*(*F*^2^) = 0.100	*w* = 1/[σ^2^(*F*_o_^2^) + (0.0516*P*)^2^ + 0.3846*P*] where *P* = (*F*_o_^2^ + 2*F*_c_^2^)/3
*S* = 1.02	(Δ/σ)_max_ < 0.001
2677 reflections	Δρ_max_ = 0.20 e Å^−^^3^
287 parameters	Δρ_min_ = −0.17 e Å^−^^3^
Primary atom site location: structure-invariant direct methods	Extinction correction: none

### Special details

**Table:**

Geometry. All e.s.d.'s (except the e.s.d. in the dihedral angle between two l.s. planes) are estimated using the full covariance matrix. The cell e.s.d.'s are taken into account individually in the estimation of e.s.d.'s in distances, angles and torsion angles; correlations between e.s.d.'s in cell parameters are only used when they are defined by crystal symmetry. An approximate (isotropic) treatment of cell e.s.d.'s is used for estimating e.s.d.'s involving l.s. planes.
Refinement. Refinement of *F*^2^ against ALL reflections. The weighted *R*-factor *wR* and goodness of fit *S* are based on *F*^2^, conventional *R*-factors *R* are based on *F*, with *F* set to zero for negative *F*^2^. The threshold expression of *F*^2^ > σ(*F*^2^) is used only for calculating *R*-factors(gt) *etc*. and is not relevant to the choice of reflections for refinement. *R*-factors based on *F*^2^ are statistically about twice as large as those based on *F*, and *R*- factors based on ALL data will be even larger.

### Fractional atomic coordinates and isotropic or equivalent isotropic displacement parameters (Å^2^)

**Table:**

	*x*	*y*	*z*	*U*_iso_*/*U*_eq_	
O1	0.9957 (2)	0.46948 (17)	0.91649 (6)	0.0407 (5)	
H10H	1.0621	0.4080	0.9120	0.061*	
O2	0.3082 (2)	0.30783 (16)	0.67021 (6)	0.0341 (4)	
H20H	0.2534	0.3094	0.6439	0.051*	
O3	0.2237 (2)	0.30076 (18)	0.56873 (6)	0.0436 (5)	
O4	0.7859 (2)	0.53134 (15)	0.59363 (6)	0.0305 (4)	
O5	0.7923 (2)	0.74167 (16)	0.59427 (6)	0.0369 (4)	
C1	0.9214 (3)	0.5459 (3)	0.78456 (8)	0.0302 (5)	
H1A	1.0060	0.5598	0.7581	0.036*	
H1B	0.8524	0.6236	0.7884	0.036*	
C2	1.0228 (3)	0.5220 (3)	0.83123 (9)	0.0342 (6)	
H2A	1.1021	0.4498	0.8266	0.041*	
H2B	1.0951	0.5962	0.8389	0.041*	
C3	0.9010 (4)	0.4954 (2)	0.87292 (8)	0.0338 (6)	
H3	0.8282	0.5718	0.8785	0.041*	
C4	0.7785 (3)	0.3879 (2)	0.86049 (9)	0.0329 (6)	
H4A	0.8471	0.3095	0.8585	0.039*	
H4B	0.6921	0.3780	0.8868	0.039*	
C5	0.6821 (3)	0.4070 (2)	0.81332 (9)	0.0291 (5)	
C6	0.5095 (3)	0.3951 (2)	0.81037 (9)	0.0329 (6)	
H6	0.4469	0.3787	0.8393	0.039*	
C7	0.4070 (3)	0.4058 (3)	0.76433 (9)	0.0349 (6)	
H7A	0.3450	0.4871	0.7638	0.042*	
H7B	0.3182	0.3385	0.7632	0.042*	
C8	0.5244 (3)	0.3962 (2)	0.71992 (8)	0.0289 (5)	
H8	0.5711	0.3089	0.7191	0.035*	
C9	0.6822 (3)	0.4840 (2)	0.72681 (8)	0.0273 (5)	
H9	0.6346	0.5680	0.7358	0.033*	
C10	0.7967 (3)	0.4390 (2)	0.76984 (8)	0.0276 (5)	
C11	0.7907 (3)	0.5023 (2)	0.68051 (8)	0.0292 (6)	
H11A	0.8714	0.5738	0.6850	0.035*	
H11B	0.8622	0.4266	0.6747	0.035*	
C12	0.6770 (3)	0.5265 (2)	0.63685 (8)	0.0284 (5)	
H12	0.6155	0.6084	0.6409	0.034*	
C13	0.5413 (3)	0.4230 (2)	0.62780 (8)	0.0282 (5)	
C14	0.4220 (3)	0.4154 (2)	0.67314 (9)	0.0283 (5)	
C15	0.3094 (3)	0.5331 (2)	0.66889 (9)	0.0324 (6)	
H15A	0.3745	0.6079	0.6798	0.039*	
H15B	0.2013	0.5250	0.6883	0.039*	
C16	0.2667 (3)	0.5414 (3)	0.61473 (9)	0.0361 (6)	
H16A	0.2616	0.6299	0.6044	0.043*	
H16B	0.1522	0.5019	0.6080	0.043*	
C17	0.4140 (3)	0.4716 (2)	0.58736 (9)	0.0311 (5)	
H17	0.4780	0.5329	0.5666	0.037*	
C18	0.6315 (3)	0.2983 (2)	0.61696 (9)	0.0324 (6)	
H18A	0.6999	0.2722	0.6451	0.049*	
H18B	0.7093	0.3083	0.5891	0.049*	
H18C	0.5431	0.2347	0.6097	0.049*	
C19	0.9048 (3)	0.3220 (2)	0.75633 (9)	0.0306 (6)	
H19A	0.9960	0.3454	0.7332	0.046*	
H19B	0.8279	0.2592	0.7418	0.046*	
H19C	0.9590	0.2873	0.7854	0.046*	
C20	0.3456 (3)	0.3660 (2)	0.55563 (9)	0.0336 (6)	
C21	0.4373 (4)	0.3454 (3)	0.50851 (9)	0.0427 (7)	
H21A	0.5622	0.3317	0.5144	0.064*	
H21B	0.4221	0.4191	0.4879	0.064*	
H21C	0.3877	0.2719	0.4924	0.064*	
C22	0.8251 (3)	0.6433 (2)	0.57417 (9)	0.0296 (5)	
C23	0.9065 (3)	0.6338 (2)	0.52566 (9)	0.0301 (6)	
C24	0.9321 (4)	0.7573 (3)	0.50011 (11)	0.0379 (6)	
H24A	1.0187	0.8073	0.5176	0.057*	
H24B	0.8209	0.8026	0.4990	0.057*	
H24C	0.9735	0.7420	0.4672	0.057*	
C25	0.9463 (3)	0.5220 (3)	0.50738 (9)	0.0344 (6)	
H25	0.9221	0.4519	0.5274	0.041*	
C26	1.0243 (4)	0.4940 (3)	0.45924 (9)	0.0412 (7)	
H26A	1.0654	0.5719	0.4444	0.062*	
H26B	0.9359	0.4553	0.4385	0.062*	
H26C	1.1230	0.4364	0.4632	0.062*	

### Atomic displacement parameters (Å^2^)

**Table:**

	*U*^11^	*U*^22^	*U*^33^	*U*^12^	*U*^13^	*U*^23^
O1	0.0448 (11)	0.0395 (11)	0.0377 (10)	0.0083 (10)	−0.0043 (9)	−0.0037 (8)
O2	0.0286 (9)	0.0326 (9)	0.0412 (9)	−0.0092 (8)	−0.0009 (8)	0.0016 (8)
O3	0.0432 (10)	0.0413 (11)	0.0463 (10)	−0.0129 (10)	−0.0051 (10)	0.0009 (9)
O4	0.0285 (8)	0.0268 (9)	0.0363 (9)	0.0004 (8)	0.0056 (8)	0.0033 (8)
O5	0.0416 (10)	0.0273 (10)	0.0417 (10)	−0.0030 (9)	0.0086 (9)	−0.0013 (8)
C1	0.0268 (12)	0.0270 (13)	0.0369 (13)	−0.0032 (11)	0.0028 (11)	−0.0010 (11)
C2	0.0316 (12)	0.0317 (14)	0.0395 (14)	−0.0052 (12)	0.0004 (12)	−0.0019 (12)
C3	0.0355 (13)	0.0312 (15)	0.0346 (13)	0.0060 (12)	−0.0026 (12)	−0.0002 (11)
C4	0.0313 (12)	0.0314 (14)	0.0360 (13)	0.0003 (12)	0.0050 (11)	0.0008 (11)
C5	0.0279 (12)	0.0221 (13)	0.0371 (13)	0.0007 (10)	0.0024 (11)	0.0005 (10)
C6	0.0299 (13)	0.0333 (14)	0.0354 (13)	−0.0004 (12)	0.0046 (11)	0.0028 (12)
C7	0.0264 (13)	0.0380 (15)	0.0403 (15)	−0.0009 (12)	0.0048 (12)	0.0024 (12)
C8	0.0227 (11)	0.0254 (13)	0.0386 (13)	−0.0004 (11)	0.0007 (11)	0.0018 (11)
C9	0.0246 (11)	0.0237 (13)	0.0337 (13)	0.0011 (10)	0.0024 (10)	0.0006 (10)
C10	0.0239 (11)	0.0243 (13)	0.0345 (13)	−0.0002 (10)	0.0027 (10)	−0.0004 (10)
C11	0.0215 (11)	0.0298 (14)	0.0362 (13)	−0.0012 (10)	0.0008 (10)	0.0020 (10)
C12	0.0252 (12)	0.0263 (13)	0.0338 (13)	0.0008 (11)	0.0036 (11)	0.0018 (11)
C13	0.0227 (11)	0.0265 (13)	0.0354 (13)	0.0016 (10)	−0.0007 (10)	0.0025 (11)
C14	0.0200 (11)	0.0269 (13)	0.0381 (13)	−0.0023 (10)	0.0037 (11)	−0.0003 (11)
C15	0.0245 (12)	0.0305 (13)	0.0422 (13)	0.0005 (11)	0.0027 (12)	−0.0007 (12)
C16	0.0327 (13)	0.0323 (14)	0.0434 (15)	0.0060 (12)	0.0001 (12)	0.0011 (12)
C17	0.0277 (11)	0.0288 (13)	0.0369 (13)	0.0004 (11)	−0.0009 (11)	0.0029 (11)
C18	0.0340 (13)	0.0260 (13)	0.0372 (13)	0.0012 (11)	−0.0004 (12)	−0.0009 (11)
C19	0.0267 (12)	0.0280 (14)	0.0372 (13)	0.0000 (11)	0.0017 (11)	0.0006 (11)
C20	0.0333 (13)	0.0289 (14)	0.0388 (14)	0.0014 (12)	−0.0067 (12)	0.0057 (11)
C21	0.0473 (17)	0.0415 (17)	0.0393 (15)	0.0039 (14)	−0.0017 (14)	−0.0023 (13)
C22	0.0238 (12)	0.0273 (13)	0.0379 (13)	−0.0017 (11)	−0.0014 (11)	0.0030 (11)
C23	0.0238 (12)	0.0337 (14)	0.0327 (13)	−0.0027 (11)	−0.0010 (11)	0.0032 (11)
C24	0.0379 (14)	0.0369 (15)	0.0388 (13)	−0.0039 (13)	0.0075 (13)	0.0026 (11)
C25	0.0318 (13)	0.0343 (15)	0.0371 (13)	0.0018 (12)	0.0005 (12)	0.0025 (12)
C26	0.0426 (15)	0.0396 (17)	0.0415 (15)	0.0064 (13)	0.0018 (13)	−0.0021 (12)

### Geometric parameters (Å, °)

**Table:**

O1—C3	1.435 (3)	C11—H11B	0.9900
O1—H10H	0.8400	C12—C13	1.538 (3)
O2—C14	1.445 (3)	C12—H12	1.0000
O2—H20H	0.8400	C13—C18	1.530 (3)
O3—C20	1.220 (3)	C13—C14	1.555 (3)
O4—C22	1.347 (3)	C13—C17	1.573 (3)
O4—C12	1.460 (3)	C14—C15	1.529 (3)
O5—C22	1.216 (3)	C15—C16	1.538 (3)
C1—C2	1.530 (3)	C15—H15A	0.9900
C1—C10	1.543 (3)	C15—H15B	0.9900
C1—H1A	0.9900	C16—C17	1.550 (3)
C1—H1B	0.9900	C16—H16A	0.9900
C2—C3	1.511 (3)	C16—H16B	0.9900
C2—H2A	0.9900	C17—C20	1.523 (4)
C2—H2B	0.9900	C17—H17	1.0000
C3—C4	1.523 (4)	C18—H18A	0.9800
C3—H3	1.0000	C18—H18B	0.9800
C4—C5	1.515 (3)	C18—H18C	0.9800
C4—H4A	0.9900	C19—H19A	0.9800
C4—H4B	0.9900	C19—H19B	0.9800
C5—C6	1.329 (3)	C19—H19C	0.9800
C5—C10	1.529 (3)	C20—C21	1.498 (4)
C6—C7	1.502 (3)	C21—H21A	0.9800
C6—H6	0.9500	C21—H21B	0.9800
C7—C8	1.527 (3)	C21—H21C	0.9800
C7—H7A	0.9900	C22—C23	1.485 (3)
C7—H7B	0.9900	C23—C25	1.333 (4)
C8—C14	1.528 (3)	C23—C24	1.511 (4)
C8—C9	1.542 (3)	C24—H24A	0.9800
C8—H8	1.0000	C24—H24B	0.9800
C9—C11	1.540 (3)	C24—H24C	0.9800
C9—C10	1.555 (3)	C25—C26	1.492 (4)
C9—H9	1.0000	C25—H25	0.9500
C10—C19	1.545 (3)	C26—H26A	0.9800
C11—C12	1.512 (3)	C26—H26B	0.9800
C11—H11A	0.9900	C26—H26C	0.9800
			
C3—O1—H10H	109.5	C12—C13—C14	107.59 (19)
C14—O2—H20H	109.5	C18—C13—C17	115.3 (2)
C22—O4—C12	119.14 (18)	C12—C13—C17	107.28 (19)
C2—C1—C10	114.4 (2)	C14—C13—C17	103.22 (18)
C2—C1—H1A	108.7	O2—C14—C8	104.46 (18)
C10—C1—H1A	108.7	O2—C14—C15	108.09 (18)
C2—C1—H1B	108.7	C8—C14—C15	117.7 (2)
C10—C1—H1B	108.7	O2—C14—C13	110.49 (19)
H1A—C1—H1B	107.6	C8—C14—C13	113.02 (17)
C3—C2—C1	111.38 (19)	C15—C14—C13	103.07 (19)
C3—C2—H2A	109.4	C14—C15—C16	104.0 (2)
C1—C2—H2A	109.4	C14—C15—H15A	111.0
C3—C2—H2B	109.4	C16—C15—H15A	111.0
C1—C2—H2B	109.4	C14—C15—H15B	111.0
H2A—C2—H2B	108.0	C16—C15—H15B	111.0
O1—C3—C2	111.6 (2)	H15A—C15—H15B	109.0
O1—C3—C4	110.8 (2)	C15—C16—C17	107.2 (2)
C2—C3—C4	110.4 (2)	C15—C16—H16A	110.3
O1—C3—H3	108.0	C17—C16—H16A	110.3
C2—C3—H3	108.0	C15—C16—H16B	110.3
C4—C3—H3	108.0	C17—C16—H16B	110.3
C5—C4—C3	113.1 (2)	H16A—C16—H16B	108.5
C5—C4—H4A	109.0	C20—C17—C16	112.9 (2)
C3—C4—H4A	109.0	C20—C17—C13	112.3 (2)
C5—C4—H4B	109.0	C16—C17—C13	105.12 (19)
C3—C4—H4B	109.0	C20—C17—H17	108.8
H4A—C4—H4B	107.8	C16—C17—H17	108.8
C6—C5—C4	121.6 (2)	C13—C17—H17	108.8
C6—C5—C10	122.9 (2)	C13—C18—H18A	109.5
C4—C5—C10	115.52 (19)	C13—C18—H18B	109.5
C5—C6—C7	124.3 (2)	H18A—C18—H18B	109.5
C5—C6—H6	117.8	C13—C18—H18C	109.5
C7—C6—H6	117.8	H18A—C18—H18C	109.5
C6—C7—C8	111.86 (19)	H18B—C18—H18C	109.5
C6—C7—H7A	109.2	C10—C19—H19A	109.5
C8—C7—H7A	109.2	C10—C19—H19B	109.5
C6—C7—H7B	109.2	H19A—C19—H19B	109.5
C8—C7—H7B	109.2	C10—C19—H19C	109.5
H7A—C7—H7B	107.9	H19A—C19—H19C	109.5
C7—C8—C14	111.91 (18)	H19B—C19—H19C	109.5
C7—C8—C9	108.7 (2)	O3—C20—C21	122.2 (2)
C14—C8—C9	115.15 (19)	O3—C20—C17	121.0 (2)
C7—C8—H8	106.9	C21—C20—C17	116.8 (2)
C14—C8—H8	106.9	C20—C21—H21A	109.5
C9—C8—H8	106.9	C20—C21—H21B	109.5
C11—C9—C8	113.34 (18)	H21A—C21—H21B	109.5
C11—C9—C10	111.96 (18)	C20—C21—H21C	109.5
C8—C9—C10	110.35 (19)	H21A—C21—H21C	109.5
C11—C9—H9	106.9	H21B—C21—H21C	109.5
C8—C9—H9	106.9	O5—C22—O4	122.6 (2)
C10—C9—H9	106.9	O5—C22—C23	124.1 (2)
C5—C10—C1	108.17 (19)	O4—C22—C23	113.2 (2)
C5—C10—C19	108.47 (19)	C25—C23—C22	120.1 (2)
C1—C10—C19	109.41 (19)	C25—C23—C24	125.1 (2)
C5—C10—C9	110.49 (19)	C22—C23—C24	114.7 (2)
C1—C10—C9	108.76 (19)	C23—C24—H24A	109.5
C19—C10—C9	111.48 (19)	C23—C24—H24B	109.5
C12—C11—C9	112.20 (18)	H24A—C24—H24B	109.5
C12—C11—H11A	109.2	C23—C24—H24C	109.5
C9—C11—H11A	109.2	H24A—C24—H24C	109.5
C12—C11—H11B	109.2	H24B—C24—H24C	109.5
C9—C11—H11B	109.2	C23—C25—C26	127.7 (2)
H11A—C11—H11B	107.9	C23—C25—H25	116.2
O4—C12—C11	109.50 (17)	C26—C25—H25	116.2
O4—C12—C13	106.10 (18)	C25—C26—H26A	109.5
C11—C12—C13	113.3 (2)	C25—C26—H26B	109.5
O4—C12—H12	109.3	H26A—C26—H26B	109.5
C11—C12—H12	109.3	C25—C26—H26C	109.5
C13—C12—H12	109.3	H26A—C26—H26C	109.5
C18—C13—C12	110.73 (19)	H26B—C26—H26C	109.5
C18—C13—C14	112.2 (2)		
			
C10—C1—C2—C3	−56.4 (3)	O4—C12—C13—C17	−69.3 (2)
C1—C2—C3—O1	178.4 (2)	C11—C12—C13—C17	170.54 (19)
C1—C2—C3—C4	54.7 (3)	C7—C8—C14—O2	−67.2 (2)
O1—C3—C4—C5	−176.92 (19)	C9—C8—C14—O2	168.12 (18)
C2—C3—C4—C5	−52.8 (3)	C7—C8—C14—C15	52.7 (3)
C3—C4—C5—C6	−128.9 (3)	C9—C8—C14—C15	−72.1 (3)
C3—C4—C5—C10	51.8 (3)	C7—C8—C14—C13	172.7 (2)
C4—C5—C6—C7	−176.5 (2)	C9—C8—C14—C13	48.0 (3)
C10—C5—C6—C7	2.7 (4)	C18—C13—C14—O2	−49.4 (2)
C5—C6—C7—C8	15.6 (4)	C12—C13—C14—O2	−171.42 (18)
C6—C7—C8—C14	−176.4 (2)	C17—C13—C14—O2	75.3 (2)
C6—C7—C8—C9	−48.1 (3)	C18—C13—C14—C8	67.3 (3)
C7—C8—C9—C11	−168.6 (2)	C12—C13—C14—C8	−54.8 (2)
C14—C8—C9—C11	−42.2 (3)	C17—C13—C14—C8	−168.0 (2)
C7—C8—C9—C10	64.9 (2)	C18—C13—C14—C15	−164.7 (2)
C14—C8—C9—C10	−168.70 (19)	C12—C13—C14—C15	73.3 (2)
C6—C5—C10—C1	131.8 (3)	C17—C13—C14—C15	−39.9 (2)
C4—C5—C10—C1	−48.9 (3)	O2—C14—C15—C16	−76.7 (2)
C6—C5—C10—C19	−109.6 (3)	C8—C14—C15—C16	165.4 (2)
C4—C5—C10—C19	69.7 (3)	C13—C14—C15—C16	40.3 (2)
C6—C5—C10—C9	12.9 (3)	C14—C15—C16—C17	−25.1 (3)
C4—C5—C10—C9	−167.9 (2)	C15—C16—C17—C20	122.9 (2)
C2—C1—C10—C5	51.3 (3)	C15—C16—C17—C13	0.2 (3)
C2—C1—C10—C19	−66.7 (3)	C18—C13—C17—C20	23.8 (3)
C2—C1—C10—C9	171.30 (19)	C12—C13—C17—C20	147.6 (2)
C11—C9—C10—C5	−173.29 (19)	C14—C13—C17—C20	−98.9 (2)
C8—C9—C10—C5	−46.0 (2)	C18—C13—C17—C16	147.0 (2)
C11—C9—C10—C1	68.1 (2)	C12—C13—C17—C16	−89.2 (2)
C8—C9—C10—C1	−164.62 (18)	C14—C13—C17—C16	24.2 (2)
C11—C9—C10—C19	−52.6 (3)	C16—C17—C20—O3	−37.5 (3)
C8—C9—C10—C19	74.7 (2)	C13—C17—C20—O3	81.1 (3)
C8—C9—C11—C12	45.5 (3)	C16—C17—C20—C21	143.6 (2)
C10—C9—C11—C12	171.1 (2)	C13—C17—C20—C21	−97.7 (2)
C22—O4—C12—C11	−102.8 (2)	C12—O4—C22—O5	9.5 (3)
C22—O4—C12—C13	134.6 (2)	C12—O4—C22—C23	−168.83 (18)
C9—C11—C12—O4	−174.75 (19)	O5—C22—C23—C25	176.5 (3)
C9—C11—C12—C13	−56.5 (3)	O4—C22—C23—C25	−5.2 (3)
O4—C12—C13—C18	57.3 (2)	O5—C22—C23—C24	−5.4 (4)
C11—C12—C13—C18	−62.9 (2)	O4—C22—C23—C24	172.9 (2)
O4—C12—C13—C14	−179.76 (17)	C22—C23—C25—C26	178.5 (2)
C11—C12—C13—C14	60.1 (2)	C24—C23—C25—C26	0.6 (4)

### Hydrogen-bond geometry (Å, °)

**Table:**

*D*—H···*A*	*D*—H	H···*A*	*D*···*A*	*D*—H···*A*
O1—H10H···O5^i^	0.84	2.11	2.941 (3)	173
O2—H20H···O3	0.84	2.10	2.886 (2)	156

Symmetry codes: (i) −*x*+2, *y*−1/2, −*z*+3/2.
